# “There could be something going wrong and I wouldn’t even know”: a qualitative study of perceptions of people with cancer about cardiovascular disease (CVD) risk and its management

**DOI:** 10.1007/s11764-023-01468-0

**Published:** 2023-09-29

**Authors:** Reegan Knowles, Emma Kemp, Michelle Miller, Bogda Koczwara

**Affiliations:** 1 College of Medicine and Public Health, Flinders University, Adelaide, South Australia Australia; 2 College of Education, Psychology, and Social Work, Flinders University, Adelaide, South Australia Australia; 3 Caring Futures Institute, Flinders University, Adelaide, South Australia Australia; 4 College of Medicine and Public Health, Flinders Medical Centre, Flinders University, Adelaide, South Australia Australia

**Keywords:** Cardiovascular disease, Cancer, People with cancer, Perceived needs

## Abstract

**Purpose:**

Despite being at higher risk, many people with cancer do not receive adequate cardiovascular disease (CVD) risk assessment or management. The purpose of this research was to examine people with cancer’s perceptions, experiences and needs regarding CVD risk factor awareness, assessment and management.

**Methods:**

We conducted 15 individual interviews to examine people with cancer’s perspectives regarding CVD care in cancer. Reflexive thematic analysis was utilised to collect and organise data into themes and to synthesise findings.

**Results:**

Fifteen people (6 males) diagnosed with diverse cancer types participated. Majority participants were not or only somewhat aware of CVD risk in cancer, but all expressed it was an important issue. A diverse range of priorities and needs for CVD care was discussed, including some participants’ prioritisation of dealing with cancer and preferred amount, type and manner of information provision and support. Websites and brochures were identified as potential solutions for optimising CVD care.

**Conclusions:**

Codesign methodology should be used to engage patients in the development of flexible, tailored resources to increase awareness of CVD risk and strategies for its management.

**Implications for Cancer Survivors:**

Perceptions of people with cancer regarding CVD care can inform new interventions that reduce the impact of CVD in cancer.

## Background

Cancer is associated with increased risk of cardiovascular disease (CVD) and resulting morbidity and mortality because of shared risk factors (e.g. smoking and age) and cardiotoxic anticancer treatments including radiotherapy and chemotherapy [1–4]. Older people are particularly vulnerable, given they have higher rates of both cancer and CVD compared to their younger counterparts [5–7]. Guidelines recommend people with cancer receive CVD risk factor assessment, surveillance and be assisted to reduce their risk [8, 9] including support to engage in healthy lifestyle behaviours, such as physical activity [10, 11] and smoking cessation [12].

Despite evidence and guidelines, many people with cancer do not receive adequate CVD risk assessment and management because of multiple reasons including clinician lack of time, resources or belief that this is not their role to do so [11, 13–15]. Older people may be even less likely to receive adequate CVD care, given there is evidence that preventive health services are delivered less frequently as age increases [15].

In previous research conducted by the authors, health care providers (HCPs) reported patient-level factors that they perceived may impact patients’ engagement in CVD care (including socioeconomic disadvantage, a fatalistic outlook and aversion to further medical intervention) [16], but to the authors’ knowledge, no research has examined perceptions of people who have been diagnosed with cancer regarding their awareness of CVD risk in cancer, barriers and enablers to engaging in CVD risk assessment and management, or preferences for improved approaches to CVD care in cancer. Therefore, the aim of this research was to examine people with cancer’s perceptions and experiences of CVD risk factor awareness, assessment and management in cancer.

## Methods

### Design

This was a qualitative study involving individual interviews with people with cancer.

### Eligibility and recruitment

People aged 18 years or older, diagnosed with any type of cancer regardless of stage or prognosis, and at any stage on the cancer continuum (e.g. during treatment, survivorship) were eligible to participate. People with cancer were recruited using a non-random, purposive sampling technique. Clinicians working at Flinders Medical Centre, a large metropolitan, tertiary referral centre in Southern Australia (oncologists and oncology nurses) and known through researchers’ existing networks, agreed to introduce the research to potential participants. A recruitment flyer and Participant Information and Consent Form were provided to potential participants by their clinician, and the contact details of patients willing to be contacted were provided to researchers. A researcher (RK) then contacted all potential participants by telephone and arranged informed consent and interviews for participants (either in person or via telephone).

### Study procedures

The same researcher (RK) conducted all semi-structured individual interviews. Participants were prompted to discuss their experiences and perceptions around CVD risk in cancer. We continued to recruit participants until we perceived adequate “information power” [17], that is, sufficient data to address the aims of the research, to provide a meaningful contribution to the research literature and to inform future research and practice, particularly in the development of a new approach to CVD risk identification and management in people with cancer. Ceasing recruitment based on reaching adequate information power aligns with the principle that original data may continue to emerge with subsequent interviews, and the decision to cease recruitment is therefore pragmatic [17].

### Data analysis

Interviews were audio-recorded and transcribed verbatim. We used NVivo (Version 1.3) [18] to assist in coding and theme development, and the construction and interpretation of findings occurred through a series of discussions between all researchers.

Reflexive thematic analysis (RTA), underpinned by a critical realism, was used to analyse data. Our analysis, as per the RTA framework [19], involved data familiarisation, coding, initial theme development, review of themes, refinement and reporting. The majority analysis was semantic and inductive, meaning the construction of knowledge was based on the data explicitly communicated by participants in their interviews. However, we also used latent analysis to identify more complex meanings which underlie participants’ responses. RTA is an approach that acknowledges the role of the researcher in the construction of knowledge derived from data collection, analysis and interpretation(19). The researchers practise reflexivity through all stages of analysis to analyse how our own perceptions and experiences influence the construction of knowledge derived from the analysis. The process of the construction of knowledge was iterative, recursive and flexible, where new data and ideas arose in subsequent interviews, through researcher discussions and drafting of results.

### Ethics

Ethics approval was granted by the Southern Adelaide Local Health Network Ethics Committee on 22 January, 2020 (HREC/19/SAC/309).

## Results

A total of 15 people (*n* = 6 male) participated in the research. Thirteen interviews were conducted via telephone, and two were conducted in-person. Six participants had been diagnosed with breast cancer, one with oesophageal cancer, one cervical cancer and one rectal cancer. Six participants did not report the type of cancer they had been diagnosed with. The interviews lasted between 10 min, 33 s and 46 min, 21 s (median = 23 min, 1 s).

**Fig. 1:**
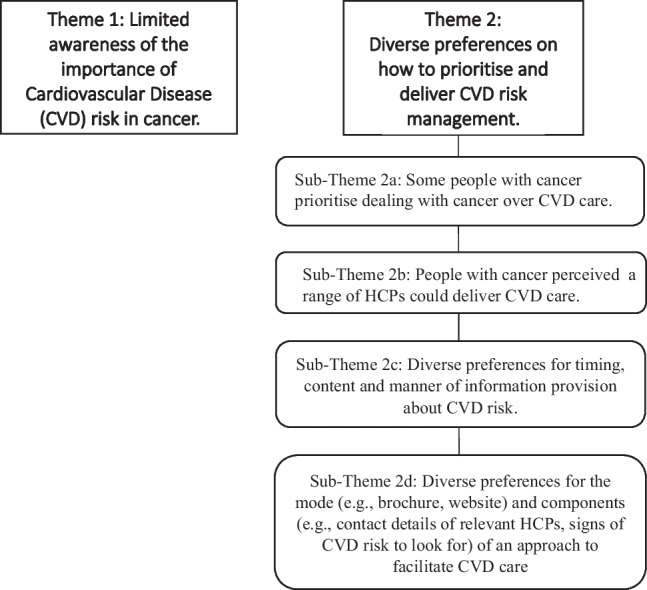
Overview of themes and sub-themes summarising people with cancer’s perceptions of CVD risk identification and management in people with cancer

The analysis identified two themes, one of which had four subthemes (Fig. 1).

### Theme 1: Limited awareness of the importance of CVD risk in cancer.

The majority of respondents indicated that they did not know CVD risk was increased in cancer and had not been told about this by anyone in their cancer care team. For example, “I don’t think I knew that it [cancer] really affected the heart.”

Others indicated that they deduced there was a risk, without having been explicitly informed by anyone in their cancer care team.

“*He [my oncologist] mentioned the word toxic chemotherapy… I knew exactly what he meant… [the oncologist told me it was] just toxic. Yeah, nothing [specific] to do with the heart. I know, it's my understanding that that that kind of chemotherapy actually destroys the heart muscles, reduces the ejection fraction.*”

Some were unsure as to whether they had been informed of increased CVD risk, querying their own capacity to absorb information at the time it was provided.

“*At the beginning I wasn’t really aware of it [CVD risk] at all. It [CVD risk] may have been mentioned to me perhaps in passing earlier on, but because you’re taking on board so many other things and you’re so concentrated on what’s happening to you with your cancer, I don’t really remember even considering it or thinking that it could be a concern back then”.*

*…“I know it’s silly, but they could have mentioned it [CVD risk], but the thing is I reckon for the first three months I didn’t hear a word that they said.”*

A minority of respondents were aware of increased CVD risk, most of whom had been informed of this by their oncologist.

“*Yeah, especially after radiation that it [CVD risk] could definitely affect me.”*

All respondents agreed increased CVD risk is an important issue and that it is important to be aware of it.

“*Oh, look, I just like to know, that’s all. There’s no reason, I just want to know what’s happening, you know?* *I don’t want to be – I want the doctor to tell me the truth about stuff like that.*”

Some participants acknowledged that informing patients about increased CVD risk could elicit negative emotions, but all but one reported they wanted to be fully informed despite this. For example, one participant said:

“*I want them to tell me [about CVD risk…irrespective of how devastating it would be…”*

Only one participant suggested they did not want to be told about CVD risk:

“*And I just don’t know that it’s fruitful to be told of all the things that might go wrong because then you almost manifest that to happen.”*

### Theme 2: Diverse preferences on how to prioritise and deliver CVD risk management*. management*

Diverse preferences for when, how and who should be involved in CVD care were discussed.

#### Sub-theme 2a: Some people with cancer prioritise dealing with cancer over CVD care.

A small number of respondents discussed they prioritised dealing with cancer over CVD care.

“*I suppose the big picture’s trying to sort the other – the cancer out* ”.

#### Sub-theme 2b: People with cancer perceived a range of HCPs could deliver CVD care.

Participants identified HCPs they perceived to be appropriate to assist them to self-manage their needs and provide CVD assessment and management, including nurses, oncologists and GPs. They provided reasons for why they had determined these HCPs suitable for this role. For example,

“*Because you can get in to see the GP usually a lot easier than you can the oncologist and if the GP’s doing ECGs and he’s checking your blood pressure and all the other general medication and issues, I think the GPs do one that actually know about it and do something about it for you.*”

“*…he’s [the GP] supposed to be the guy that takes care of me and the oncologist is the guy who looks after the cancer itself. So, this heart disease, it’s not part of the oncology treatment, it’s a separate issue. So, therefore, your GP should be the guy that’s says, alright, cancer – we can start expecting heart disease as well.*”

“*these guys [nurses] see everyone every day constantly and they don’t back down, they come up again and try and help support you.*”

One participant communicated that a range of HCPs could effectively guide them to manage aspects of their CVD care:

“*Everyone seems to be fairly knowledgeable [about everything health-related]… so I really wouldn’t mind who [guided me]*.”

#### Sub-theme 2c: Diverse preferences for timing, amount and manner of information provision about CVD risk

Some respondents felt the timing of CVD risk assessment and management was important and could impact their engagement with CVD care. Respondents had specific ideas about *when* would be appropriate, and this varied from participant to participant. For example, some participants would prefer to engage in CVD risk management after treatment:

“*[At the end of treatment] I would have a capacity to think okay, now I'm getting back into the swing of life… I think I would have the mental capacity to process the information…”*

In contrast, another participant expressed a preference for information to be provided earlier in the cancer continuum:

“*I’d rather know all of it [cancer and associated risks including CVD risk] in one go, or like spaced out so I kind of absorb it and then I know the next bit and the next bit, but I want it all relatively soon*.”

There were also diverse preferences for *how much* information should be provided, suggesting that flexible and tailored information provision approaches are needed. For example,

“*I’m the sort of person that needs to know basically what’s going on, I don’t need to get into the really nitty gritty, but I like to know and then I try and process it and then I try and get on with it…*”

“*Sometimes you can get bombarded* *with stuff you don’t need to know.*”

Other participants reported a preference for being provided with a comprehensive amount of information about CVD risk. For example,

“*I’d rather have all the information and then I can read back through it if I need to, if I feel like I need to…”*

“*I want to know all the information. Yeah. Yeah, definitely. I think that you can't advocate for your health for yourself if you're not informed.”*

Although respondents reported they want to be informed of CVD risk in cancer, many communicated that the *way in which information is provided* should be carefully considered. For example:

“D*efinitely [CVD risk information should be] presented in a way that doesn’t scare you more because you’re already so scared.”*

To illustrate the importance of the manner of CVD care, one participant gave an example of a negative experience of how they received care previously when they were admitted into hospital (after being diagnosed with COVID-19):

“*One or two doctors need to get some bedside manner... but I probably would have if they had taken it differently – and it might sound a bit precious, but I do think if you can just do it a different way, just you’re not as severe, do you want to be resuscitated or what?”*

#### Sub-theme 2d: Diverse preferences for the mode (e.g. brochure, website) and components (e.g. contact details of relevant HCPs, signs of CVD risk to look for) of an approach to facilitate CVD care.

A range of suggestions was provided by respondents regarding how CVD risk information could be provided and how people with cancer can be supported to undergo CVD risk assessment and management. A brochure and website for providing CVD risk information and guidance were suggested.

“*Website is pretty good too. You can go on that website and go and read what to expect.”*

Another respondent discussed their preference for support groups in which participants discuss and assist each other to navigate aspects of cancer (including CVD risk).

“*So, I’d like to be able to have a resource or the opportunity to regularly check in with others going through similar treatments and processes and I suppose there’s a warmth attached to that, talking to people that are going through similar experiences, but there’s also a real knowledge base asking really targeted individual questions in the hope that, yeah, okay, someone else has had that same experience and this is what was advised to me, so it can either then build your confidence that everything’s probably okay.”*

Other respondents indicated they would prefer they were informed about CVD risk and supported to manage their risk through direct interactions with HCPs. For example,

“*Speaking to somebody [about CVD risk] really, but also reading, definitely where you can ask questions.”*

Respondents also discussed specific components of an optimal approach to reduce the impact of CVD in cancer including:

- Provision of information about CVD risk in cancer:
  “*I want to know all the information. Yeah. Yeah, definitely. I think that you can't advocate for your health for yourself if you're not informed”.*
- List of CVD risk-related symptoms/signs the person with cancer should be aware of:
  “*I mean, there could be something going wrong and I wouldn’t even know. Sometimes I’m hopping around and think oh, okay, fine, you know? So, you just don’t know what level of damage has happened or is happening and so, I don’t know, if there is some sort of a measure…”*
- List of HCPs/services that could be contacted to assist with CVD issues:
  “*I think it’s…where to go to get advice if you feel you need to...”*
- Self-management support:
  “*In the end, my health is my responsibility. I’m the one that needs to study up on these things and to understand how my body’s going to respond to the cancer and I’m the one who needs to [deal with it]*.”
- Guidance/support for CVD risk reduction (including behaviour change):
  *i“If [I was advised that] a specific type of training will reduce your risk of heart damage…, I’d try and put them into my training…”*

## Discussion

This study of perceptions of people diagnosed with cancer regarding the CVD risk assessment and management shows that majority of respondents were not fully aware of CVD risk in cancer but once aware, they perceived it to be an important issue and had diverse preferences on how to prioritise and deliver CVD risk management.

The majority of participants in our study were relatively unaware of increased CVD risk in cancer, is concerning given guidelines which highlight the importance of CVD risk identification, monitoring and management in cancer [8, 9]. We also found that majority of people with cancer want to know about CVD risk, which aligns with evidence that providing patients with health care information can improve a patient’s feelings of control, decrease anxiety and even improve clinical outcomes [20]. In contrast, some respondents expressed they may have been told about CVD risk but had not “absorbed” the information due to “information overload” and/or prioritisation of coping with their cancer diagnosis and treatment. This aligns with concerns of cancer care providers that people with cancer may be burdened by receiving information about CVD risk whilst dealing with cancer [16]. Our findings provide an example of the concept termed by Jensen et al. “cancer information overload” (CIO), where up to three quarters of people with cancer have reported being overwhelmed by information [21]. Given CIO can induce fatalistic thinking and reduced engagement in positive health behaviour [21], it is important to consider how to meet the expressed needs of patients to be health-informed, whilst also reducing the risk of CIO. Thoughtful consideration of how, how much and when information provision occurs may be able to achieve the balance between these opposing concepts.

We identified diverse priorities and preferences for CVD risk identification and management in cancer. This is unsurprising given the lack of awareness of CVD risk of majority participants, which implies limited (previous) consideration of the possibility and practicality of potential solutions. It is also important to acknowledge that diversity of health care preferences likely reflects differences in people (e.g. demographics, attitude/outlook and personalities) and their disease (e.g. cancer type and stage in continuum). Hence, our research highlights the need for a new approach to CVD risk identification and management that is holistic, multi-pronged, flexible and tailored to individual needs and preferences. The importance of consumer engagement in health research and services is well-established [22], including recognition that the preferences and ideas of consumers (in this case people affected by cancer and cancer care providers) should inform new care approaches [22] through codesign methodology. The diversity of preferences identified in our research necessitates a comprehensive codesign approach (e.g. multiple rounds of discussion) to facilitate the development of ideas and preferences leading to approach design.

Examining perceptions of people affected by cancer regarding CVD risk in cancer facilitates comparison with the authors’ previous research involving cancer care providers [16]. An important difference between the perceptions of people with cancer and those who provide cancer care was perceived barriers to CVD. Cancer care providers identified multiple barriers including perceived conflicts with role identity and lack of time and training, whilst people with cancer did not identify barriers (16). This may reflect patients’ lack of awareness of CVD risk in cancer, meaning they likely have not considered potential barriers, and may perhaps also reflect health care consumers’ trust in the Australian health care system. Meanwhile, HCPs are aware of CVD risk and that care is not always adequate, so they are more likely to have considered potential reasons for this. In contrast, an important similarity between people affected by cancer and HCPs’ perceptions about CVD in cancer emerged, in that both had diverse ideas for solutions to optimise CVD care. This agreement reiterates the importance of future approaches to CVD care in cancer being flexible and individualised and emphasises the need for authentic and meaningful contribution of a broad range of stakeholders in the conceptualisation, design and implementation of CVD interventions for this population.

### Strengths and limitations

This is the first study to examine people with cancer’s perceptions and experiences of CVD in cancer. There were important limitations of our research. All participants were recruited from one health care setting and were of the same ethnicity, limiting extrapolation of findings to other contexts. We did not collect data from participants regarding cancer stage or phase on the cancer continuum (e.g. during active treatment, survivorship), nor the presence of existing CVD or CVD risk factors, meaning comparisons of perspectives according to demographics was not possible.

## Conclusion

People with cancer tend to have limited awareness of CVD risk and have diverse preferences regarding how to prioritise and manage it. Future research should employ codesign methodology to engage patients in the development of flexible, tailored resources to increase awareness of CVD risk and strategies for management.

## Data Availability

The data collected for this research is not available given the risk that the data may lead to the identfiication of participants.
